# Prevalence of disease in 549 older cats undergoing health screening at two United Kingdom-based veterinary clinics

**DOI:** 10.1093/jvimsj/aalag157

**Published:** 2026-08-06

**Authors:** Jason P Bestwick, Jonathan Elliott, Isabel R Orriss, Nicola Lötter, Rebecca F Geddes

**Affiliations:** Clinical Science and Services, Royal Veterinary College, Royal College Street, London NW1 0TU, United Kingdom; Comparative Biomedical Sciences, Royal Veterinary College, Royal College Street, London NW1 0TU, United Kingdom; Comparative Biomedical Sciences, Royal Veterinary College, Royal College Street, London NW1 0TU, United Kingdom; Comparative Biomedical Sciences, Royal Veterinary College, Royal College Street, London NW1 0TU, United Kingdom; Clinical Science and Services, Royal Veterinary College, Royal College Street, London NW1 0TU, United Kingdom

**Keywords:** aging, chronic renal failure, feline, primary care practice, urinary tract

## Abstract

**Background:**

Risk of some diseases of cats increases with age, including chronic kidney disease (CKD) and hyperthyroidism, but clinical signs can be dismissed by caregivers as age-related changes.

**Hypothesis/Objectives:**

Quantify the prevalence of disease and other major clinical findings in older cats.

**Animals:**

Data from cats (≥8 years old) that underwent routine health screening at 2 United Kingdom (UK)-based primary care veterinary clinics over a 52-month period (October 2, 2018 to February 14, 2023) were analyzed.

**Methods:**

At screening visits, history, physical examination, and blood tests for plasma biochemistry with or without plasma total thyroxine concentration were performed. When possible, systolic blood pressure measurement (97.1% of cases; Doppler method) and urinalysis (41.3% of cases; 98.2% by cystocentesis; and bacterial culture if indicated based on sediment examination) were performed.

**Results:**

A total of 549 cats were screened with a median age of 13.5 (IQR: 11.3-15.8) years old. Diagnoses of hyperthyroidism, azotemic CKD, and hypertension were made in 89/464 (19.2%; 95% CI, 15.9%-23.0%), 62/542 (11.4%; 95% CI, 9.0%-14.4%), and 37/524 (7.1%; 95% CI, 5.2%-9.6%) of cats, respectively. Severe dental disease was present in 32/522 (6.1%; 95% CI, 4.4%-8.5%) and bacteriuria was identified in 22/227 (9.7%; 95% CI, 6.5%-14.2%) of urine samples. Other diagnoses included suspected neoplasia (8 cats), diabetes mellitus (8 cats), and suspected primary gastrointestinal disease (4 cats).

**Conclusions and clinical importance:**

Routine medical health screening in older cats is useful in detecting diseases, many of which would benefit from prompt veterinary intervention. These results can assist veterinarians in explaining the value of health screening to cat caregivers.

## Introduction

The American Association of Feline Practitioners, based on expert panelist opinion, currently recommends examination of healthy cats aged 10-15 years biannually and every 4 months for cats > 15 years old, with laboratory diagnostic testing (including hematology, serum biochemistry, and urinalysis) and measurement of systolic blood pressure (SBP) advised at least annually and at increased frequency with advancing age.^1^ The aim of health screening is to facilitate the detection of common diseases of older cats, including chronic kidney disease (CKD), hyperthyroidism, and hypertension, which appear to be of increased prevalence with advancing age.^2–6^ Other conditions (eg, neoplasia, dental disease, and cardiomyopathy) may also be diagnosed through the routine diagnostic procedures carried out as part of health screening.^1^^,^^7–9^

However, data around the health screening recommendations provided by veterinarians, their uptake by cat caregivers, suitable intervals between screening events, and the utility of the diagnostic tests performed is challenging to obtain and likely variable among both individual veterinarians and practices. In the United Kingdom (UK), individual cats are less likely to be seen by veterinary practices than dogs.^10^ This difference could be a reflection of dogs experiencing a higher disease burden than cats, but could also indicate that cats are less likely to display clinical signs, or more likely to have them dismissed or unrecognized by caregivers. Supporting the latter hypothesis, in 206 middle-aged (7-10 years old) cats assessed by a university-based aging cat research clinic, although caregivers did report physical (53%), behavioral (47%), eating (41%), and activity changes (40%), many more cats (88%) were found to have evidence of at least 1 disease or abnormality.^7^ In a more recent study investigating the utility of repeated health screening in apparently healthy mature (7-10 years old) and senior (>10 years old) cats over a 2-year period in another university-based clinic, 21% of cats were not considered healthy at baseline and a further 28% of mature and 54% of senior cats initially considered healthy developed a new disease during follow-up.^9^

Our aim was to report disease prevalence and other findings that warrant veterinary intervention in a large number of older cats presented for prospective health screening in a primary care practice (PCP) setting in the UK, using well-defined diagnostic criteria.

## Materials and methods

### Study design

Cats were health screened as part of a longitudinal observational project examining the health of aging cats (Royal Veterinary College Ethics and Welfare Committee [RVC-EWC] approved—URN20131258E/URN202322253). Data are from cats assessed for inclusion in a prospective diet trial (RVC-EWC approved—URN 201818453/URN 202321963) during a 52-month period (October 2, 2018 to February 14, 2023).

Cats were assessed by the RVC Aging Cat Clinic (London, UK) at 2 PCPs: Beaumont Sainsbury Animal Hospital (university-owned) and Bow People’s Dispensary for Sick Animals Pet Hospital (charity-associated).

Cats newly presented during the study period and cats previously examined, lacking overt disease, and returning for re-assessment (qualifying every 6 months) were eligible for inclusion. Before screening, informed caregiver consent was obtained (Figure S1; Supplementary Appendix A).

### Study population and procedures

Cats were ≥ 8 years old and presented for routine health screening, and were excluded if they had prior diagnoses requiring long-term monitoring or management.

During screening, a clinical history was taken and a physical examination was performed (Supplementary Appendix A). Dental scoring was performed using a standardized subjective individual grading system for tartar and gingivitis: 0 = none, 1 = minimal, 2 = mild, 3 = moderate, and 4 = severe. Blood samples were taken for biochemistry (IDEXX Laboratories, Wetherby, UK), PCV, and plasma total protein concentrations.

Plasma total thyroxine (TT4) concentration (Siemens IMMULITE TT4 assay, Siemens Healthineers, Llanberis, Gwynedd, North Wales, UK) was measured if not evaluated in the previous 12 months or if there was clinical suspicion of hyperthyroidism. If the TT4 concentration was in the upper portion of the reference interval (RI) of 3.1-4.3 μg/dL (40-55 nmol/L), clinicians advised repeated measurement (typically after 8 weeks) or immediate measurement of plasma thyroid-stimulating hormone using the canine assay (cTSH; Siemens IMMULITE Canine TSH assay, Siemens Healthineers, Llanberis, Gwynedd, North Wales, UK) based upon clinical suspicion.

Systolic blood pressure (SBP) was measured using the Doppler method (Supplementary Appendix A; Doppler Flow Detector Model 811-B, Parks Medical Electronics, Aloha, OR, USA). If SBP was ≥ 160 mmHg, retinal examination (ClearView 1 or ClearView 2, Optibrand, Fort Collins, CO, USA) was performed to assess for hypertensive chorioretinopathy.^11^ If present, antihypertensives were prescribed. If not present, it was recommended that cats be returned for repeated SBP measurement to assess for persistence. Urine was collected for urinalysis with bacterial culture if microscopic bacteriuria was present.

Criteria used to diagnose diseases and describe clinical findings are detailed in Table 1.

**Table 1:** Criteria used to define the diagnosis of a disease or finding in older cats undergoing health screening.

**Diagnosis/Finding**	**Criteria**
**Azotemic CKD**	Plasma creatinine concentration > 2 mg/dL (>177 μmol/L) with concurrent USG < 1.035; or, plasma creatinine > 2 mg/dL (>177 μmol/L) at the screening and consecutive follow-up visit recommended for approximately 2-4 weeks later.
**Hyperthyroidism**	Plasma TT4 > 4.3 μg/dL (>55 nmol/L); or, TT4 3.1-4.3 μg/dL (40-55 nmol/L) and cTSH < 0.03 ng/mL.
**Hypertension**	Mean SBP ≥ 160 mmHg with evidence of TOD (hypertensive chorioretinopathy) or repeatable mean SBP ≥ 160 mmHg on re-examination recommended for 1-2 weeks later.
**Other diagnoses/findings:**	
**Increased ALT activity**	Plasma ALT activity ≥ 2× RI (≥120 U/L)
**Bacteriuria**	Bacteria observed on urine sediment examination and positive urine culture
**Dental disease**	
**Presence of tartar**	Tartar score > 0
**Presence of gingivitis**	Gingivitis score > 0
**Severe dental disease**	Tartar or gingivitis score = 4
**Suspected neoplasia**	Palpable or visible mass lesion
**Diabetes mellitus**	Hyperglycemia and glucosuria with or without increased serum fructosamine concentration
**Suspected primary GI disease**	Chronic vomiting or diarrhea without an identified cause

Abbreviations: ALT, alanine transferase; CKD, chronic kidney disease; cTSH, canine thyroid-stimulating hormone; GI, gastrointestinal; RI, reference interval; SBP, systolic blood pressure; TOD, target organ damage; TT4, total thyroxine; USG, urine specific gravity.

### Statistical analyses

Data were compiled and descriptive statistics performed using Microsoft Excel (Version 16.101.1). The 95% CI for disease prevalence was calculated using Wilson’s method.^12^

## Results

### Overall study population

A total of 549 eligible cats were health screened during the 52-month study period. The median age was 13.5 years (IQR, 11.3-15.8). Two hundred and sixty-eight (268/549; 48.8%) of the cats were male (2 intact, 266 neutered) and 281/549 (51.2%) were female (1 intact, 280 spayed). Seventy-eight percent (428/549) of the cats were domestic shorthair, 68/549 (12.4%) were domestic longhair, 43/549 (7.8%) were purebred, 7/549 (1.3%) were purebred crosses, and 3/549 (0.5%) did not have their breed recorded. Median body weight was 4.0 kg (IQR, 3.3-4.7).

For the majority of cats (468/549; 85.2%), the health screen was their first visit to the clinic. The remaining cats (81/549; 14.8%) had been examined ≥ 6 months previously and deemed healthy. Health screening was performed by 10 clinicians and a single veterinary nurse during the study period.

### Health screening findings

#### Hyperthyroidism

**Figure 1:**
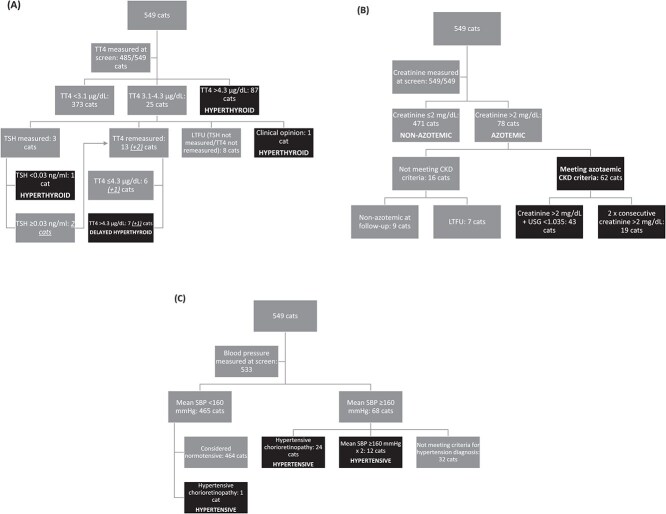
Flow diagrams showing the routes to diagnosis (or non-diagnosis) in 549 health screened older cats for: (A) hyperthyroidism, (B) azotemic CKD, and (C) hypertension. Abbreviations: CKD = chronic kidney disease; cTSH = canine thyroid-stimulating hormone; LTFU = lost to follow-up; SBP = systolic blood pressure; TT4 = total thyroxine.

At the time of health screening, hyperthyroidism was diagnosed in 19.2% of cats (89/464; Figure 1A). This estimate does not include cases without a plasma TT4 measurement at the time of health screening (*n* = 64). It does include cats diagnosed with hyperthyroidism on the basis of a plasma TT4 concentration > 4.3 μg/dL (>55 nmol/L; *n* = 87) and cats with hyperthyroidism excluded (plasma TT4 < 3.1 μg/dL [<40 nmol/L]; *n* = 373). For cats with plasma TT4 concentrations between 3.1 and 4.3 μg/dL (40-55 nmol/L; *n* = 25), it includes those that had cTSH measured (*n* = 3), resulting in either a diagnosis (*n* = 1) or non-diagnosis of hyperthyroidism (*n* = 2), as well as a single cat considered hyperthyroid outside of the diagnostic criteria specified in Table 1. Regarding this cat, medical treatment commenced (PO thiamazole) without measurement of plasma cTSH, on the basis of compatible clinical signs and suspected sick euthyroid syndrome secondary to concurrent azotemic CKD. Treatment response was compatible with the diagnosis.

Of the remaining 21 cats with a plasma TT4 concentration between 3.1 and 4.3 μg/dL (40-55 nmol/L) and excluded from the prevalence estimate, 8 were lost to follow-up (ie, did not have cTSH or follow-up TT4 measured) and 13 had a later TT4 measurement available for review (Figure 1A): 6 had plasma TT4 concentrations ≤ 4.3 μg/dL (≤55 nmol/L; median follow-up 102 days; IQR, 19-215 days) and 7 received a delayed diagnosis of hyperthyroidism (TT4 > 4.3 μg/dL [>55 nmol/L]; median follow-up, 161 days; IQR, 98-186 days). In addition, the 2 cats with a plasma TT4 concentration between 3.1 and 4.3 μg/dL (40-55 nmol/L) and cTSH ≥ 0.03 ng/mL at the time of health screening also had later plasma TT4 concentrations available for review: one continued to have a TT4 concentration ≤ 4.3 μg/dL (≤55 nmol/L; 440 days after screening) and the other was diagnosed with hyperthyroidism (TT4 > 4.3 μg/dL [>55 nmol/L]; 196 days after screening).

#### Azotemic CKD

All cats had plasma creatinine concentration measured at the time of health screening; 227/549 (41.3%) cats had urinalysis simultaneously performed.

Overall, 62/542 (11.4%) cats were diagnosed with azotemic CKD (Figure 1B): 43/62 (69.4%) based on a concurrent plasma creatinine concentration > 2 mg/dL (>177 μmol/L) and USG < 1.035 and 19/62 (30.6%) based on plasma creatinine concentration > 2 mg/dL (>177 μmol/L) on 2 consecutive visits at least 2 weeks apart. An additional 16 cats were azotemic without a concurrent urine sample at the time of health screening, but were not diagnosed with azotemic CKD because 9/16 (56.3%) were non-azotemic at a subsequent visit and 7/16 (43.8%) were lost to follow-up. These latter cats were excluded from the overall azotemic CKD estimate because of missing follow-up data required to ascertain disease status.

Plasma symmetric dimethylarginine (SDMA) concentrations were available for 62/62 and 486/487 cats diagnosed with and without azotemic CKD, respectively. For cats diagnosed with azotemic CKD, SDMA was above the RI (>14 μg/dL) in 46/62 (74.2%) cats and ≥ 18 μg/dL in 30/62 (48.4%) cats. For cats not diagnosed with azotemic CKD, SDMA was above the RI (>14 μg/dL) in 57/486 (11.7%) cats and ≥ 18 μg/dL in 13/486 (2.7%) cats.

#### Hypertension

In total, 533/549 (97.1%) cats had SBP measured at the time of their health screening visit and 37/524 (7.1%) were diagnosed as hypertensive (Figure 1C). One (1/37) cat was diagnosed as hypertensive outside of the diagnostic criteria specified in Table 1, with a mean SBP < 160 mmHg (148 mmHg), because of evidence of retinal edema (bullae) and tortuous retinal vessels in both eyes.

Of the 533 cats that had SBP measured, 32 cats had SBP of ≥ 160 mmHg but were not diagnosed with or treated for hypertension. Short-term follow-up was not available for 9 of these cats but, for all 9 cats, retinal examination was normal at the time of health screening. These 9 cats were excluded from the overall hypertension estimate because of missing follow-up data required to ascertain disease status. For the remaining 23 cats, mean SBP was < 160 mmHg at follow-up and the initial mean SBP of ≥ 160 mmHg was suspected to be a result of situational hypertension on the basis of lack of evidence for hypertensive chorioretinopathy (some had vessel tortuosity suspected but as a lone finding) and inconsistency of the finding on repeated examination.

#### Concurrent hyperthyroidism, azotemic CKD, and hypertension

**Figure 2:**
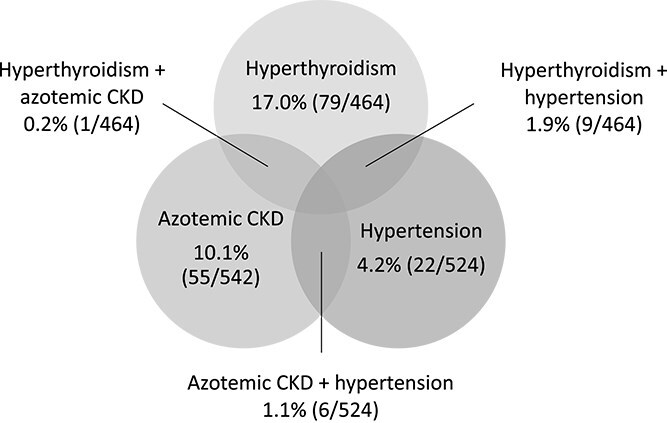
Venn diagram showing concurrent prevalence of the 3 most commonly diagnosed medical conditions of 549 older cats undergoing health screening. Abbreviation: CKD = chronic kidney disease.

Sixteen cats were diagnosed with more than 1 of the 3 most common medical conditions (hyperthyroidism, azotemic CKD, and hypertension; Figure 2).

#### Bacteriuria

Of the 549 cats, 227/549 (41.3%) had urine collected at the time of screening: 4/227 voided and 223/227 collected by palpation-guided cystocentesis. Bacteria were observed on sediment examination in 37/227 (16.3%) and urine samples therefore were submitted for bacterial culture and antibiotic sensitivity testing. Bacterial cultures were positive in 23 of 37 (62.2%) but one positive result was considered a contaminant based on the bacteria cultured (*Psychrobacter faecalis* and *Bacillus* spp.) and a voided sample being utilized (Table 2).

**Table 2:** Frequency of bacterial isolates cultured from urine samples obtained from health screened older cats. All samples were acquired by palpation-guided cystocentesis. One positive culture is excluded because of suspected contamination (voided sample; *Psychrobacter faecalis* and *Bacillus* spp. cultured).

**Bacteria cultured**	**Number of cats (*n*/22)**
***Escherichia coli* (*E coli*) only**	7
***Enterococcus faecalis* (*E faecalis*) only**	7
***E coli* and *E faecalis***	2
***E coli*, *E faecalis,* and *Hafnia* spp.**	1
***E coli* and *Klebsiella oxytoca***	1
***Staphylococcus***	
**spp.**	2
***pseudintermedius***	1
***felis***	1

**Figure 3:**
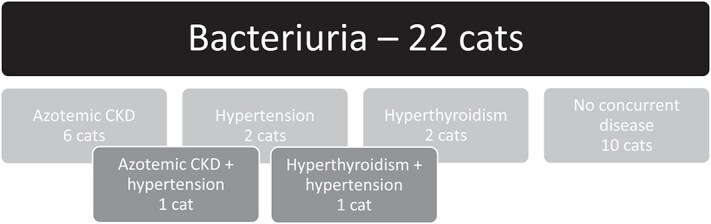
Diagram showing concurrent disease diagnoses of the 22 cats with bacteriuria. Abbreviation: CKD = chronic kidney disease.

Of the 22 cats (3 male neutered, 19 female spayed) with positive urine bacterial cultures considered not to be contaminants, *Escherichia coli* was the most common bacterial isolate either as the sole agent or in combination with other bacteria (11/22; 50.0%) and CKD was the most common concurrent disease diagnosed (7/22; 31.8%; Figure 3).

#### Other diagnoses and findings

Diabetes mellitus was diagnosed in 8/549 (1.5%) cats: 5 based on hyperglycemia and glucosuria alone and 3 based on hyperglycemia, glucosuria, and a compatible serum fructosamine concentration (at the clinician’s discretion). None of these cats had azotemic CKD, hyperthyroidism, hypertension, or bacteriuria (urine samples acquired by cystocentesis from 5/8 cats; 2/5 submitted for bacterial culture, both negative) concurrently diagnosed.

Neoplasia was suspected in 8/549 (1.5%) cats based on physical examination: 5 abdominal masses of uncertain origin, 1 bilateral renal enlargement (lymphoma suspected), 1 oral mass, and 1 facial mass. Of the 5 cats with abdominal masses of uncertain origin, one was also concurrently diagnosed as hypertensive and another as hyperthyroid.

Four cats (4/549; 0.7%) were suspected to have primary gastrointestinal (GI) disease on the basis of caregiver-reported chronic vomiting or diarrhea or both in the absence of an overt extra-GI cause on investigations performed for the health screen.

An increase in alanine aminotransferase (ALT) activity ≥ 2× the RI (≥120 U/L) was found in 82/549 (14.9%) of cats. Of these, the increase was attributable to hyperthyroidism in 53/82 (64.6%) cats and diabetes mellitus in 4/82 (4.9%) cats. The cause of the ALT increase was unclear in the remaining 25 cats (25/82; 30.5%).

**Figure 4:**
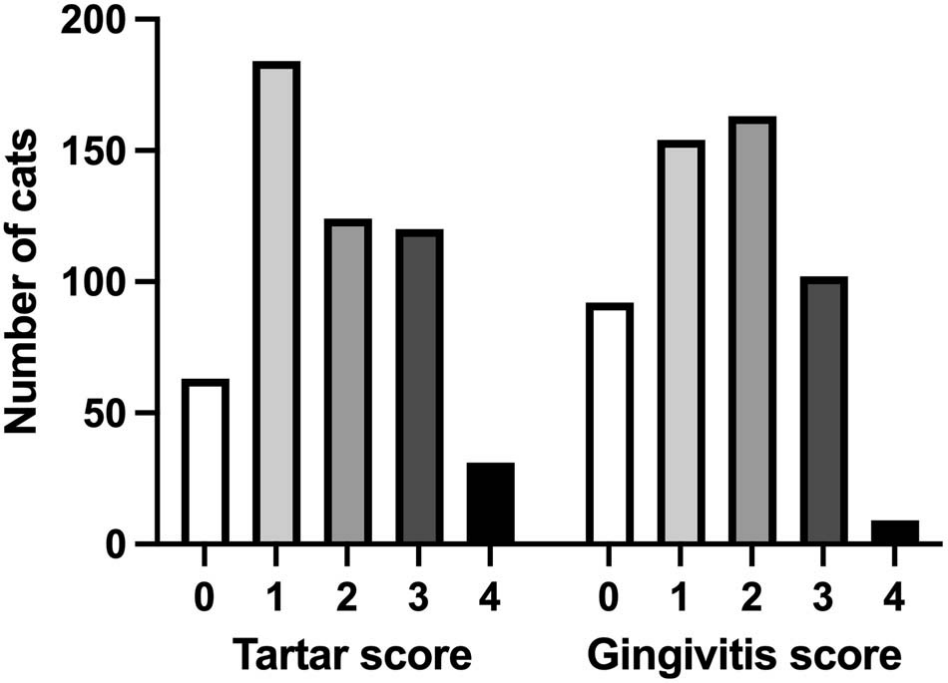
A bar chart showing the frequency with which each tartar and gingivitis score (0 = no tartar or gingivitis, 1 = minimal tartar or gingivitis, 2 = mild tartar or gingivitis, 3 = moderate tartar or gingivitis, and 4 = severe tartar or gingivitis) was assigned in 549 health screened older cats.

Five-hundred and twenty-two of 549 cats had a tartar score, and 520/549 cats had a gingivitis score, available for review (Figure 4). Of these, 459/522 (87.9%) and 428/520 (82.3%) had some degree of tartar and gingivitis (tartar or gingivitis score > 0), respectively, and among them 32/522 (6.1%) had severe tartar or gingivitis or both (tartar or gingivitis score = 4).

One cat was found to be moderately anemic (PCV 17%) without an identifiable cause. Further investigations were advised but follow-up was not available.

#### Prevalence of diagnoses and findings

Derived from the results above, Table 3 shows the calculated prevalence and 95% CI for major diseases and clinical findings from the 549 cats undergoing health screening during a 52-month period (October 2, 2018 to February 14, 2023).

**Table 3:** Prevalence and 95% CIs for major diseases and clinical findings from the 549 older cats undergoing health screening during a 52-month period (October 2, 2018 to February 14, 2023).

**Diagnosis/Finding**	**No. cats diagnosed**	**Prevalence (%) [95% CI]**
**Hyperthyroidism**	89/464^a^	19.2 [15.9-23.0]
**Azotemic CKD**	62/542^b^	11.4 [9.0-14.4]
**Hypertension**	37/524^c^	7.1 [5.2-9.6]
**Other diagnoses/findings:**		
**Increased ALT activity ≥ 2× RI**	82/549^d^	14.9 [12.2-18.2]
**Bacteriuria**	22/227^e^	9.7 [6.5-14.2]
**Dental disease**		
**Tartar**	459/522^f^	87.9 [84.9-90.5]
**Gingivitis**	428/520^f^	82.3 [78.8-85.3]
**Severe dental disease**	32/522^f^	6.1 [4.4-8.5]
**Suspected neoplasia**	8/549^g^	1.5 [0.7-2.8]
**Diabetes mellitus**	8/549^h^	1.5 [0.7-2.8]
**Suspected primary GI disease**	4/549^i^	0.7 [0.3-1.9]
**Moderate or severe anemia of unknown cause**	1/549^j^	0.2 [0.0-1.0]

a Plasma TT4 measured in 485/549 cats. Twenty-one cats with a plasma TT4 concentration between 3.1 and 4.3 μg/dL (40-55 nmol/L) without concurrent measurement of cTSH were either lost to follow-up (*n* = 8) or had delayed diagnosis (*n* = 7) or non-diagnosis of hyperthyroidism (*n* = 6) and were excluded from the prevalence estimate.

b Plasma creatinine and USG measured in 549/549 and 227/549 cats, respectively. Seven cats that were azotemic but without concurrent urinalysis and lacking follow-up were excluded from the prevalence estimate.

c SBP measured in 533/549 cats. Nine cats with a mean SBP ≥ 160 mmHg at health screening had an absence of hypertensive chorioretinopathy but lacked follow-up blood pressure measurements, and were excluded from the prevalence estimate.

d Plasma ALT measured in 549/549 cats.

e Urinalysis performed in 227/549 cats; bacteria observed on sediment examination in 37/227 samples and sent for bacterial culture.

f Tartar and gingivitis scores recorded for 522/549 and 520/549 cats, respectively.

g Physical examination performed in 549/549 cats.

h Glucosuria assessed for in all cats with an available urine sample (227/549); blood glucose measured in 344/549 cats, including all cats without an available urinalysis.

i Clinical history taken, physical examination, and plasma biochemistry performed for 549/549 cats.

j PCV and total solids measured in 549/549 cats.

Abbreviations: ALT, alanine transferase; CKD, chronic kidney disease; cTSH, canine thyroid-stimulating hormone; GI, gastrointestinal; RI, reference interval; SBP, systolic blood pressure; TT4, total thyroxine; USG, urine specific gravity.

#### Findings in previously examined cats

Eighty-one cats (81/549) had been examined previously at the clinic (and found to be healthy) and were presented again for health screening during the period of the study. Sixty of these cats (60/81) were presented ≤ 24 months after their previous examination. Regarding these cats, 4/59 (6.8%) were diagnosed with azotemic CKD at the time of health screening. At the prior examination, all 4 cats had plasma creatinine concentration ≤ 2 mg/dL (≤177 μmol/L) and 2 cats had plasma SDMA concentration > 14 μg/dL, one of these had a concentration ≥ 18 μg/dL. Hypertension was diagnosed in 2/58 (3.4%) cats and individual cases of hyperthyroidism (1/33; 3.0%) and diabetes mellitus (1/60; 1.7%) also were diagnosed, all in separate cats. None of these 60 cats had a positive urine culture and 9/60 cats had an ALT ≥ 2× RI, including the hyperthyroid, 2 hypertensive, and 1 of the azotemic CKD cats. One cat (1/60; 1.7%) had severe tartar or gingivitis or both (tartar or gingivitis score = 4).

## Discussion

We report the prevalence of disease and major clinical findings for a large population of health-screened cats in a PCP setting. Forty-two percent (231/549) of cats aged 8 years and older had ≥ 1 condition that might benefit from veterinary intervention or further investigation, including hyperthyroidism, azotemic CKD and hypertension, severe dental disease, diabetes mellitus, suspected neoplasia, suspected primary GI disease, increased ALT activity (≥2× RI), bacteriuria, and moderate to severe anemia of unknown cause. Some of these diseases are progressive and have management options shown to improve outcomes or ameliorate disease sequelae.^13–17^ Hence, routine health screening of older cats (≥8 years old) detects a high frequency of previously unrecognized abnormalities or conditions and, as a consequence may facilitate earlier intervention, which has been associated with improved outcomes.

Moreover, in a subpopulation of cats that were examined ≤ 24 months before their current health screening visit (and found to have an absence of overt disease at this prior assessment), diagnoses were still made. The prevalence of disease and notable findings within this subpopulation is less than that found in the entire population of health-screened cats. This finding was expected because these cats had known veterinary assessments that previously excluded overt disease, compared with those visiting the clinic for the first time with variable degrees of veterinary contact. However, the fact that diseases potentially benefiting from veterinary intervention were still found within this subpopulation supports the value of repeated health screening in cats ≥ 8 years old, as also was demonstrated in a recent study examining cats ≥ 7 years old in Belgium.^9^

Hyperthyroidism was the most common medical condition identified in our study, with a prevalence of 19.2%. This percentage falls within the wide prevalence rates for hyperthyroidism in cats reported previously (0%-21.1%),^2^^,^^7–9^^,^^18–26^ but interestingly is more than twice the prevalence of 8.7% reported for cats ≥ 10 years in a large-scale study using UK primary care practice (PCP) data from the VetCompass Animal Surveillance Project.^2^ This disparity likely demonstrates the effect of ascertainment bias, namely, the difference between performing free-of-charge prospective health screening in our study vs evaluating prevalence in a multicenter PCP setting without standardized diagnostic and inclusion criteria where many cats will not have been screened for the disease. The former possibly allows for increased detection of disease compared to situations in which cats are most likely presented only when clinical signs have been detected by a caregiver. As might be expected, measurement of TT4 in asymptomatic cats could allow for earlier disease diagnosis and intervention before overt clinical signs arise (eg, cachexia, cardiovascular disease). Previous studies that have reported the occurrence of hyperthyroidism in apparently healthy cats, reported prevalences of 0%-5.5%, which is much lower than in our study.^7–9^^,^^19^^,^^26^ This difference could be related to the younger cat populations utilized in prior studies or increasing disease prevalence over time,^27^ but also could be a result of different diagnostic and inclusion criteria as well as geographic location. An earlier study performed at the same clinic as our current study reported a baseline prevalence rate of hyperthyroidism of 5.5% in health-screened cats ≥ 9 years old.^19^ This finding may suggest that the frequency of hyperthyroidism diagnosis in this UK-based PCP population has increased between the 2 study periods (2004-2006 compared to 2018-2023).

Azotemic CKD was diagnosed in 11.4% of the 549 cats screened in our study. No attempt was made to confirm a diagnosis of CKD in non-azotemic cats, but 11.7% of non-azotemic cats in our study had a plasma SDMA concentration above the RI, which may indicate early CKD. This 11.4% prevalence of azotemic CKD is consistent with 3 other studies (2 from the UK and 1 from Belgium) that reported on the health screening of older cats and described prevalences of 10.1%, 10.6%, and 7.7% for azotemia, International Renal Interest Society (IRIS) stage 2 CKD and IRIS stage ≥ 2 CKD, respectively.^7^^,^^9^^,^^19^ Interestingly, plasma SDMA concentrations were within the RI for 25.8% of the cats diagnosed with azotemic CKD in our current study. Whereas all of our azotemic cats were IRIS stage 2 or higher based on their plasma creatinine concentrations, 51.6% of them had SDMA concentrations < 18 μg/dL, consistent with IRIS stage 1. Additionally, of the 4 cats that developed azotemic CKD after a prior assessment at the clinic ≤ 24 months earlier, only 2 had plasma SDMA concentrations above the RI (14 μg/dL) at the earlier examination. These findings were unexpected given that SDMA reportedly increases above the RI earlier than creatinine does in 81% of cats ultimately diagnosed with azotemic CKD.^28^ However, a recent study examining alterations in gut-derived uremic toxins before the onset of azotemic CKD in cats found that SDMA remained within the IRIS stage 1 interval (<18 μg/dL) for all 22 cats in the 6 months before developing azotemic CKD, and although SDMA IRIS stages at the point of azotemic CKD diagnosis were not reported, the median SDMA concentration was 13.4 μg/dL (ie, still within the RI).^29^ It therefore appears that many cats diagnosed with azotemic CKD can have a discrepancy between their assigned IRIS stages based on plasma creatinine and SDMA concentrations.

We identified hypertension in 7.1% of the cats screened, which is within the range of prevalences (2.8%-12.7%) reported in other health-screened populations of older cats.^5^^,^^7^^,^^9^^,^^19^ No underlying cause of hypertension was identified in the majority of our cases (56.8%; 21/37 cats; all 21 cats had a plasma biochemistry panel performed including measurement of plasma creatinine, SDMA, and TT4 concentrations, and 9/21 had urinalysis results). In the remaining cats (43.2%; 16/37 cats), the most common cause of potential secondary hypertension was hyperthyroidism (9/37 cats) followed by azotemic CKD (6/37 cats), and 1 cat had an abdominal mass (1/37 cats). This predominance of hypertension without an overt cause identified on the health screening tests performed is an unanticipated observation given that previous studies report that idiopathic hypertension only accounts for 13%-23% of hypertensive cats (ie, most cats have secondary hypertension).^30–32^ This finding, however, may be attributable to differences in tests performed to screen for underlying disease and SBP thresholds used to diagnose hypertension among studies, resulting in disparate study cohorts. A mean SBP cut-off of 160 mmHg was used in our study, based on the American College of Veterinary Internal Medicine (ACVIM) consensus statement on systemic hypertension,^33^ compared with 170 or 175 mmHg in earlier studies.^30–32^ The hypertensive cats in our study also were older than those in previous studies, possibly leading to the expectation that the prevalence of underlying conditions, and therefore secondary hypertension, would be higher in our population. However, in humans, although essential (idiopathic) hypertension is more common, the prevalence of secondary hypertension is higher in younger patients compared with the entire population.^34^ A lack of screening for primary hyperaldosteronism and, as previously mentioned, testing for non-azotemic CKD as potential causes of secondary hypertension are limiting factors for interpreting these data. Previous studies share these limitations and longitudinal follow-up would be required to assess if some cases of primary hypertension might in fact have an underlying cause that has not yet emerged or requires persistent documentation of mild clinicopathological abnormalities for diagnosis (eg, non-azotemic CKD). Interestingly, plasma SDMA concentrations were above the RI (>14 μg/dL) in 4/21 (19.0%) cats with hypertension and no underlying cause identified, and ≥ 18 μg/dL in 2 of these.

Bacteriuria was identified in 9.7% of cats that had urinalysis. In our protocol, urine culture was only performed if bacteria were observed on urinary sediment examination. Recently, a low prevalence (3.4%) of positive urine cultures with inactive sediment was reported in dogs.^35^ Therefore, although our methodology may have resulted in a slight underreporting of bacteriuria prevalence, the effect is expected to be minimal. Unfortunately, based on available clinical records, it was not possible for the majority of cats to determine if a urinary tract infection (UTI) or subclinical bacteriuria was present, if the cat was symptomatic or asymptomatic, or if treatment was appropriate or effective as defined by recent guidelines.^36^ More than 50% of cats with positive urine cultures had concurrent hyperthyroidism, azotemic CKD, hypertension, or some combination of these (most commonly CKD). Hyperthyroidism and CKD are predisposing factors for bacterial UTIs and subclinical bacteriuria in cats.^37–40^

In addition to hyperthyroidism, azotemic CKD, hypertension and bacteriuria, several other diseases, and important clinical findings were detected on routine health screening in our study. These included diabetes mellitus, neoplasia, and suspected primary GI disease. Diabetes mellitus was diagnosed in 1.5% of cats, a much higher prevalence than the 0.4%-0.6% reported previously in diverse populations of cats in the UK.^41^^,^^42^ We hypothesize that this higher prevalence could be in part related to the age of the respective study populations because one of the previous studies reported increased disease risk in cats > 6 years old. However, for diabetes mellitus and primary GI disease, the observed prevalences in our study are surprising considering these conditions are expected to be associated with clinical signs and that cats were presented for routine health screening rather than veterinary attention being sought sooner for an unwell cat. A possible reason for this finding includes a potential lack of recognition of illness by caregivers. This situation reflects a real-world population where cats may not only display clinical signs inconsistently and be variably presented to a veterinarian. Several factors might account for this finding, including disease severity and rate of progression, cat and caregiver lifestyle (eg, indoor, outdoor, or semi-feral cat and level of caregiver and cat interaction), and availability and cost of veterinary care.^43^^,^^44^

Dental disease, specifically gingivitis and tartar accumulation were present in the majority of cats, and 6.1% had severe tartar or gingivitis or both that would have resulted in a recommendation for further dental assessment, including examination, dental cleaning with or without tooth extractions under general anesthesia. As found here, dental disease consistently ranks among the 2 most common findings on physical examination of health-screened older cats^5^^,^^7–9^^,^^19^^,^^45^; in one study, it was associated with a matted coat and increased sleeping, potentially suggesting the presence of chronic oral pain.^7^ Because a complete oral examination cannot routinely be performed in conscious cats and dental radiographs were not obtained, it is likely that the number of cats affected by periodontal disease and that would benefit from dental procedures is higher than estimated by the detection of severe gingivitis and tartar accumulation in our study.^46^

The ALT activity was increased ≥ 2× the RI in 14.9% of cats, but the majority (69.5%) of these increases could be explained by the concurrent diagnosis of either hyperthyroidism or diabetes mellitus. In the remaining 30.5% of cats, the cause of the increased ALT activity could not be determined but could have indicated primary hepatic disease (eg, cholangitis-cholangiohepatitis syndrome or undetected neoplasia) or an undiscovered secondary cause (eg, subclinical GI disease or pancreatitis).

Our study had some limitations. In a similar way that caregivers’ ability to recognize and report illness will have impacted the study population, a subpopulation of cats will have been examined with caregivers motivated enough to seek health screening and enroll their cats into a prospective research study. Caregivers also may have been motivated to actively withhold information about their cats’ histories that could have excluded them from the free-of-charge assessments. This factor may have been somewhat ameliorated with cats recruited from the charity practice, because some caregivers already would be eligible for free pet health care. Our study used data from cats that were health screened for the purpose of identifying eligibility for a prospective diet trial. This feature could have introduced bias because the study population and processes were not explicitly designed to answer secondary questions about the utility of health screening. However, despite this limitation, some benefits may have been derived from recruitment criteria that were equally appropriate for both studies (eg, ≥8 years old, absence of chronic disease diagnoses) and the standardized clinical protocol and diagnostic criteria that were implemented. The fact that cats with pre-existing chronic conditions requiring long-term monitoring and management were excluded likely underestimates the prevalence of overall disease. However, it seems probable that older cats with ongoing conditions are more likely to have regular veterinary visits and therefore have comorbidities detected, whereas our study provides insight into a population of cats considered well by caregivers yet, as observed, a proportion are affected by disease. Of note, our study reports the value of health screening in a recruited and specific population (cats ≥ 8 years old) and prevalence estimates cannot be extrapolated to the general cat population. For some conditions, the denominator population used to calculate prevalence was adjusted (decreased) to account for cats where it could not be determined if a disease was present because of the absence of data at the time of health screening or required follow-up. Examples include azotemic patients for which urinalysis results or follow-up plasma creatinine concentration were not available (azotemic CKD), cats that had their plasma TT4 concentration measured in the 12 months before health screening rather than at the time of screening (hyperthyroidism), and cats with mean SBP ≥ 160 mmHg without evidence for hypertensive chorioretinopathy and not presented for follow-up blood pressure measurement. Depending on whether or not these cats had the disease in question, prevalence could have been overestimated or underestimated. Likewise, 2 cases (1 hyperthyroid and 1 hypertensive cat) were diagnosed outside of the stated diagnostic criteria but based upon reasonable real-world clinical reasoning.

In conclusion, routine clinical examination and diagnostic tests identified conditions requiring veterinary intervention or treatment in 42.1% of 549 cats 8 years or older in our study. These data support the need for health screening in aging cats and emphasize the importance of a veterinary examination to detect health conditions of concern. Veterinarians can utilize this information to make evidence-based recommendations on the utility and need for health screening in older cats.

## Supplementary Material

Supplementary_material_aalag157

## Off-label antimicrobial declaration

The authors declare no off-label use of antimicrobials.

## Institutional animal care and use committee or other approval declaration

Approved by the Royal Veterinary College Ethics and Welfare Committee (RVC-EWC): URN 20131258E/URN 202322253 and URN 201818453/URN 202321963.

## Abbreviations

ALT: alanine aminotransferase
cTSH: canine thyroid-stimulating hormone
CKD: chronic kidney disease
GI: gastrointestinal
PCP: primary care practice
RI: reference interval
SBP: systolic blood pressure
SDMA: symmetric dimethylarginine
TT4: total thyroxine
USG: urine specific gravity
UTI: urinary tract infection

## References

[1] Ray M, Carney HC, Boynton B, et al. 2021 AAFP feline senior care guidelines. J Feline Med Surg. 2021;23:613-638. 10.1177/1098612x211021538 34167339 PMC10812122

[2] Stephens MJ, Neill DGO, Church DB, McGreevy PD, Thomson PC, Brodbelt DC. Feline hyperthyroidism reported in primary-care veterinary practices in England: prevalence, associated factors and spatial distribution. Vet Rec. 2014;175:458. 10.1136/vr.102431 25028466

[3] Brown CA, Elliott J, Schmiedt CW, Brown SA. Chronic kidney disease in aged cats: clinical features, morphology, and proposed pathogeneses. Vet Pathol. 2016;53:309-326. 10.1177/0300985815622975 26869151

[4] Bijsmans ES, Jepson RE, Chang YM, Syme HM, Elliott J. Changes in systolic blood pressure over time in healthy cats and cats with chronic kidney disease. J Vet Intern Med. 2015;29:855-861. 10.1111/jvim.12600 25917326 PMC4895402

[5] Jepson RE, Brodbelt D, Vallance C, Syme HM, Elliott J. Evaluation of predictors of the development of azotemia in cats. J Vet Intern Med. 2009;23:806-813. 10.1111/j.1939-1676.2009.0339.x 19566846

[6] Bodey AR, Sansom J. Epidemiological study of blood pressure in domestic cats. J Small Anim Pract. 1998;39:567-573. 10.1111/j.1748-5827.1998.tb03710.x 9888110

[7] Dowgray N, Pinchbeck G, Eyre K, Biourge V, Comerford E, German AJ. Aging in cats: owner observations and clinical finding in 206 mature cats at enrolment to the cat prospective aging and welfare study. Front Vet Sci. 2022;9:859041. 10.3389/fvets.2022.859041 35445099 PMC9014291

[8] Paepe D, Verjans G, Duchateau L, Piron K, Ghys L, Daminet S. Routine health screening: findings in apparently healthy middle-aged and old cats. J Feline Med Surg. 2012;15:8-19. 10.1177/1098612x12464628 PMC10816488 23254237

[9] Mortier F, Daminet S, Marynissen S, Smets P, Paepe D. Value of repeated health screening in 259 apparently healthy mature adult and senior cats followed for 2 years. J Vet Intern Med. 2024;38:2089-2098. 10.1111/jvim.17138 38967102 PMC11256131

[10] Sánchez-Vizcaíno F, Noble PJM, Jones PH, et al. Demographics of dogs, cats, and rabbits attending veterinary practices in Great Britain as recorded in their electronic health records. BMC Vet Res. 2017;13:218. 10.1186/s12917-017-1138-9 28693574 PMC5504643

[11] Carter J . Hypertensive ocular disease in cats: a guide to fundic lesions to facilitate early diagnosis. J Feline Med Surg. 2019;21:35-45. 10.1177/1098612x18818668 30763152 PMC10814178

[12] Wilson EB . Probable inference, the law of succession, and statistical inference. J Am Stat Assoc. 1927;22:209-212. 10.1080/01621459.1927.10502953

[13] Elliott J, Rawlings JM, Markwell PJ, Barber PJ. Survival of cats with naturally occurring chronic renal failure: effect of dietary management. J Small Anim Pract. 2000;41:235-242. 10.1111/j.1748-5827.2000.tb03932.x 10879400

[14] Young WM, Zheng C, Davidson MG, Westermeyer HD. Visual outcome in cats with hypertensive chorioretinopathy. Vet Ophthalmol. 2019;22:161-167. 10.1111/vop.12575 29667738

[15] Taylor SS, Sparkes AH, Briscoe K, et al. ISFM consensus guidelines on the diagnosis and management of hypertension in cats. J Feline Med Surg. 2017;19:288-303. 10.1177/1098612x17693500 28245741 PMC11119534

[16] Reynolds BS, Lefebvre HP. Feline CKD: pathophysiology and risk factors—what do we know? J Feline Med Surg. 2013;15:3-14. 10.1177/1098612x13495234 23999182 PMC10816689

[17] Carney HC, Ward CR, Bailey SJ, et al. 2016 AAFP guidelines for the management of feline hyperthyroidism. J Feline Med Surg. 2016;18:400-416. 10.1177/1098612x16643252 27143042 PMC11132203

[18] Miyamoto T, Miyata I, Kurobane K, et al. Prevalence of feline hyperthyroidism in Osaka and the Chugoku region. J Jpn Vet Méd Assoc. 2011;55:289. 10.12935/jvma1951.55.289

[19] Wakeling J, Elliott J, Syme H. Evaluation of predictors for the diagnosis of hyperthyroidism in cats. J Vet Intern Med. 2011;25:1057-1065. 10.1111/j.1939-1676.2011.00790.x 21985139

[20] Köhler I, Ballhausen BD, Stockhaus C, Hartmann K, Wehner A. Prevalence of and risk factors for feline hyperthyroidism among a clinic population in southern Germany. Tierarztliche Prax Ausg K Kleintiere Heimtiere. 2015;44:149-157. 10.15654/tpk-150590 26902958

[21] Bree L, Gallagher BA, Shiel RE, Mooney CT. Prevalence and risk factors for hyperthyroidism in Irish cats from the greater Dublin area. Ir Vet J. 2018;71:2. 10.1186/s13620-017-0113-x 29372047 PMC5769238

[22] Domínguez AP, Tostado RS, Bernabe LF, Corredor AP, Prat JP. Prevalence of feline hyperthyroidism in a laboratory-based sample of 27,888 cats in Spain. J Feline Med Surg. 2024;26:1098612X241303304. 10.1177/1098612x241303304 PMC11672385 39713975

[23] O’Neill DG, Gunn-Moore D, Sorrell S, et al. Commonly diagnosed disorders in domestic cats in the UK and their associations with sex and age. J Feline Med Surg. 2023;25:1098612X231155016. 10.1177/1098612x231155016 PMC10812063 36852509

[24] Wet CSD, Mooney CT, Thompson PN, Schoeman JP. Prevalence of and risk factors for feline hyperthyroidism in Hong Kong. J Feline Med Surg. 2008;11:315-321. 10.1016/j.jfms.2008.08.001 18848795 PMC10911463

[25] O’Neill DG, Church DB, McGreevy PD, Thomson PC, Brodbelt DC. Prevalence of disorders recorded in cats attending primary-care veterinary practices in England. Vet J. 2014;202:286-291. 10.1016/j.tvjl.2014.08.004 25178688

[26] Dell’Osa D, Jaensch S. Prevalence of clinicopathological changes in healthy middle-aged dogs and cats presenting to veterinary practices for routine procedures. Aust Vet J. 2016;94:317-323. 10.1111/avj.12481 27569834

[27] Peterson M . Hyperthyroidism in cats. J Feline Med Surg. 2012;14:804-818. 10.1177/1098612x12464462 23087006 PMC11112171

[28] Hall JA, Yerramilli M, Obare E, Yerramilli M, Jewell DE. Comparison of serum concentrations of symmetric dimethylarginine and creatinine as kidney function biomarkers in cats with chronic kidney disease. J Vet Intern Med. 2014;28:1676-1683. 10.1111/jvim.12445 25231385 PMC4895610

[29] Mulders LV, Broecke EV, Paepe ED, Mortier F, Vanhaecke L, Daminet S. Alterations in gut-derived uremic toxins before the onset of azotemic chronic kidney disease in cats. J Vet Intern Med. 2025;39:e17289. 10.1111/jvim.17289 39739435 PMC11683462

[30] Elliott J, Barber PJ, Syme HM, Rawlings JM, Markwell PJ. Feline hypertension: clinical findings and response to antihypertensive treatment in 30 cases. J Small Anim Pract. 2001;42:122-129. 10.1111/j.1748-5827.2001.tb02008.x 11303854

[31] Maggio F, DeFrancesco TC, Atkins CE, Pizzirani S, Gilger BC, Davidson MG. Ocular lesions associated with systemic hypertension in cats: 69 cases (1985–1998). J Am Vet Méd Assoc. 2000;217:695-702. 10.2460/javma.2000.217.695 10976302

[32] Jepson RE, Elliott J, Brodbelt D, Syme HM. Effect of control of systolic blood pressure on survival in cats with systemic hypertension. J Vet Intern Med. 2007;21:402-409. 10.1111/j.1939-1676.2007.tb02982.x 17552443

[33] Acierno MJ, Brown S, Coleman AE, et al. ACVIM consensus statement: guidelines for the identification, evaluation, and management of systemic hypertension in dogs and cats. J Vet Intern Med. 2018;32:1803-1822. 10.1111/jvim.15331 30353952 PMC6271319

[34] de Freminville JB, Gardini M, Cremer A, et al. Prevalence and risk factors for secondary hypertension in young adults. Hypertension. 2024;81:2340-2349. 10.1161/hypertensionaha.124.22753 39297209

[35] Strachan NA, Hales EN, Fischer JR. Prevalence of positive urine culture in the presence of inactive urine sediment in 1049 urine samples from dogs. J Vet Intern Med. 2022;36:629-633. 10.1111/jvim.16378 35108434 PMC8965268

[36] Taylor S, Boysen S, Buffington T, et al. 2025 iCatCare consensus guidelines on the diagnosis and management of lower urinary tract diseases in cats. J Feline Med Surg. 2025;27:1098612X241309176. 10.1177/1098612x241309176 PMC11816079 39935081

[37] Teichmann-Knorrn S, Reese S, Wolf G, Hartmann K, Dorsch R. Prevalence of feline urinary tract pathogens and antimicrobial resistance over five years. Vet Rec. 2018;183:21. 10.1136/vr.104440 29622684

[38] Mayer-Roenne B, Goldstein RE, Erb HN. Urinary tract infections in cats with hyperthyroidism, diabetes mellitus and chronic kidney disease. J Feline Med Surg. 2006;9:124-132. 10.1016/j.jfms.2006.09.004 17088093 PMC10832742

[39] Dorsch R, von Vopelius-Feldt C, Wolf G, Mueller RS, Straubinger RK, Hartmann K. Urinary tract infections in cats. Prevalence of comorbidities and bacterial species, and determination of antimicrobial susceptibility to commonly used antimicrobial agents. Tierarztliche Prax Ausg K Kleintiere Heimtiere. 2016;44:227-236. 10.15654/tpk-150604 27278117

[40] White JD, Stevenson M, Malik R, Snow D, Norris JM. Urinary tract infections in cats with chronic kidney disease. J Feline Med Surg. 2013;15:459-465. 10.1177/1098612x12469522 23232285 PMC10816307

[41] O’Neill DG, Gostelow R, Orme C, et al. Epidemiology of diabetes mellitus among 193,435 cats attending primary-care veterinary practices in England. J Vet Intern Med. 2016;30:964-972. 10.1111/jvim.14365 27353396 PMC5094533

[42] McCann TM, Simpson KE, Shaw DJ, Butt JA, Gunn-Moore DA. Feline diabetes mellitus in the UK: the prevalence within an insured cat population and a questionnaire-based putative risk factor analysis. J Feline Med Surg. 2007;9:289-299. 10.1016/j.jfms.2007.02.001 17392005 PMC10822632

[43] Ma GC, McLeod LJ, Zito SJ. Characteristics of cat semi-owners. J Feline Med Surg. 2023;25:1098612X231194225. 10.1177/1098612x231194225 PMC10812031 37751179

[44] Cats Protection . CATS Report: Cats and Their Stats UK. 2025. Accessed November 5, 2025. https://www.cats.org.uk/media/onnnmi1h/cats-report-uk-2025_digital.pdf

[45] Finch NC, Syme HM, Elliott J. Risk factors for development of chronic kidney disease in cats. J Vet Intern Med. 2016;30:602-610. 10.1111/jvim.13917 26948860 PMC4864943

[46] Perry R, Tutt C. Periodontal disease in cats: back to basics—with an eye on the future. J Feline Med Surg. 2015;17:45-65. 10.1177/1098612x14560099 25527493 PMC11383102

