# Supplementary material for: Prevalence of disease in 549 older cats undergoing health screening at two United Kingdom-based veterinary clinics
Source: J Vet Intern Med. 2026 Aug 6;40(4):aalag157. doi: 10.1093/jvimsj/aalag157 (PMC13446506; doi:10.1093/jvimsj/aalag157)
Supplement: Supplementary file 1 [file supplementary_material_aalag157.zip › EDIT_Supplementary Figure 1.docx]

**Supplementary Figure 1:** Information sheet and consent form for the longitudinal observational project examining the health of aging cats.

|  |  |
| --- | --- |
