# Supplementary material for: Prevalence of disease in 549 older cats undergoing health screening at two United Kingdom-based veterinary clinics
Source: J Vet Intern Med. 2026 Aug 6;40(4):aalag157. doi: 10.1093/jvimsj/aalag157 (PMC13446506; doi:10.1093/jvimsj/aalag157)
Supplement: Supplementary file 1 [file supplementary_material_aalag157.zip › appendix.docx]

**SUPPLEMENTARY APPENDIX A:** Additional Methods

**2.1 Study design**

During the study period, caregivers were invited to bring their cats for health screening via posters and flyers placed within the practice buildings, direct invitation when attending for routine health care (e.g. booster vaccination, anti-parasitic treatment) and, in the case of returning cats, scheduled appointment reminders sent by e-mail.

**2.2 Study population and procedures**

Diagnostic procedures were performed and diagnoses made using a standardized written protocol devised by senior members of the clinic, which included members board-certified in small animal internal medicine (diplomates of the European College of Veterinary Internal Medicine, the American College of Veterinary Internal Medicine, or both). All clinicians (veterinarians undertaking postgraduate research training) performing health screening were trained in the clinical protocols.

Systolic blood pressure measurement was performed using the Doppler method (average of 5 readings; Doppler Flow Detector Model 811-B, Parks Medical Electronics, Aloha, Oregon, US), after a period of acclimatization and before any other procedures. The same equipment and devices were used across sites and all cats were restrained by the same veterinary nurse but clinicians performing the readings varied. Cats had their antebrachium circumference measured and, if <7.5 cm, a blood pressure cuff with a width of 2.5 cm was used; if ≥7.5 cm, then a 3.3 cm width blood pressure cuff was used. Cats were allowed to adopt a natural body position, with the cuff applied to a thoracic limb, and the cat in sternal recumbency or sitting. If sitting, where possible, SBP measurements were taken with the limb raised such that the transducer was level with the heart. If SBP was ≥160mmHg, a retinal examination was performed using a retinal camera (ClearView 1 or ClearView 2, Optibrand, Fort Collins, Colorado, US) to assess for evidence of hypertensive chorioretinopathy (i.e. retinal edema [bullae], retinal hemorrhage, retinal detachment, retinal vessel tortuosity).^1^ If hypertensive chorioretinopathy was present, anti-hypertensive medication was prescribed. If not, caregivers were advised to return the cats for repeated blood pressure measurement to assess for persistence of increased SBP.

Urine samples were collected primarily by palpation-guided cystocentesis and the following tests performed: urine specific gravity (USG) measured by refractometer (Master-SUR/Nα Clinical Refractometer, Atago, Tokyo, Japan), urine dipstick analysis (Combur 9 Test Urinalysis Strips, Roche, Basel, Switzerland) and sediment examination with bacterial culture if indicated by the presence of bacteria on urinary sediment examination. If a urine sample was not obtained from a cat to allow assessment of glucosuria, a point-of-care spot blood glucose measurement was performed.

**References**

1. Carter J. Hypertensive ocular disease in cats: A guide to fundic lesions to facilitate early diagnosis. J Feline Med Surg. 2019;21(1):35–45. doi:10.1177/1098612x18818668 PubMed PMID: 30763152; PubMed Central PMCID: PMC10814178.
